# Gut Microbiota, Its Role in Induction of Alzheimer’s Disease Pathology, and Possible Therapeutic Interventions: Special Focus on Anthocyanins

**DOI:** 10.3390/cells9040853

**Published:** 2020-04-01

**Authors:** Muhammad Sohail Khan, Muhammad Ikram, Jun Sung Park, Tae Ju Park, Myeong Ok Kim

**Affiliations:** 1 Division of Applied Life Science (BK 21), College of Natural Sciences, Gyeongsang National University, Jinju 52828, Korea; Sohail.bannu@gnu.ac.kr (M.S.K.); qazafi417@gnu.ac.kr (M.I.); jsp@gnu.ac.kr (J.S.P.); 2 Paul O’Gorman Leukaemia Research, Centre Institute of Cancer, Sciences University of Glasgow, 0747 657 5394 Glasgow, UK; 2358860P@student.gla.ac.uk

**Keywords:** natural polyflavonoids, gut dysbiosis, systemic inflammation, neuroinflammation, memory impairment, Alzheimer’s disease

## Abstract

The human gut is a safe environment for several microbes that are symbiotic and important for the wellbeing of human health. However, studies on gut microbiota in different animals have suggested that changes in the composition and structure of these microbes may promote gut inflammation by releasing inflammatory cytokines and lipopolysaccharides, gut-wall leakage, and may affect systemic inflammatory and immune mechanisms that are important for the normal functioning of the body. There are many factors that aid in the gut’s dysbiosis and neuroinflammation, including high stress levels, lack of sleep, fatty and processed foods, and the prolonged use of antibiotics. These neurotoxic mechanisms of dysbiosis may increase susceptibility to Alzheimer’s disease (AD) and other neurodegenerative conditions. Therefore, studies have recently been conducted to tackle AD-like conditions by specifically targeting gut microbes that need further elucidation. It was suggested that gut dyshomeostasis may be regulated by using available options, including the use of flavonoids such as anthocyanins, and restriction of the use of high-fatty-acid-containing food. In this review, we summarize the gut microbiota, factors promoting it, and possible therapeutic interventions especially focused on the therapeutic potential of natural dietary polyflavonoid anthocyanins. Our study strongly suggests that gut dysbiosis and systemic inflammation are critically involved in the development of neurodegenerative disorders, and the natural intake of these flavonoids may provide new therapeutic opportunities for preclinical or clinical studies.

## 1. Introduction

The microbial population living in human and animal intestines is termed as the gut microbiota (formerly called gut flora). Gut microbiota are trillions of micro-organisms, including at least 1000 different species of known bacteria with more than three million genes [1]. The interaction between human health and the gut microbiota is well-documented [2]. The gut microbiota comprises two phyla, *Bacteroidetes* and *Firmicutes*. At the embryonic stage, the gut microbiota appears disorganized, while by the age of three, it starts to look similar to adult flora [3]. Several main microbiota functions have been explored, including the synthesis of amino acids and vitamins that play a major role in the circulation of steroid molecules (including bile acids and sex hormones), boosting the immune system and production of different bioactive compounds [4,5].

In this comprehensive review, we summarize the current research, highlighting the role of the gut microbiota in the pathogenesis of neuroinflammation and AD-like pathologies. The literature search was comprehensive, including both published and unpublished articles. We documented the search process, kept track of the decisions that were made for each article, and screened and extracted the conclusions. Moreover, we summarized all current research from more than 350 research and review articles, showing the interaction between gut microbiota and neurocognitive function. In summary, our findings suggest that gut microbiota have a significant impact on the pathogenesis of Alzheimer’s disease, which may be significantly delayed with the administration of anthocyanin.

### 1.1. Normal Gut-Microbiota Functions in Human Body

Colonic bacteria can ferment complex carbohydrate-yielding short-chain fatty acids (SCFAs) in which propionate, butyrate, and acetate are predominantly found in the gastrointestinal tract (GI), with a ratio of 1:1:3, respectively, performing different cellular functions, such as gene expression, chemotaxis, differentiation, and proliferation. Acetate is generated by most gut anaerobes, whereas propionate and butyrate are produced by different classes of gut bacteria following distinct molecular pathways. Butyrate is generated from carbohydrates via the acetoacetyl–CoA and glycolytic pathway, whereas propionate is formed from two pathways, the succinate or propanediol pathway [6,7,8,9,10,11].

Propionate gluconeogenesis can be stimulated by the liver, while acetate and butyrate are lipogenic. Propionate and butyrate are histone deacetylase (HDAC) inhibitors, which epigenetically modulate the expression of certain important genes. SCFAs confer a key role in the regulation of immunity and inflammatory conditions. They also affect the release of cytokines, for example, by activating the release of interleukin IL-18, that is involved in the restoration and preservation of the epithelial structure. SCFAs were shown to regulate appetite and energy intake via various mechanisms [12,13,14,15,16,17,18,19].

GI microbes are also helpful in the synthesis of pivotal vitamins that cannot be produced by the host organisms. Vitamin B12, which is crucial for the body, is produced by lactic acid bacteria and is not synthesized by animals and plants. Other vitamins, such as folate that regulates the metabolic processes of the hosts, including DNA synthesis and repair, are produced by *Bifidobacteria*. A major immune shortage shown by germfree animals is the lack of CD4+ T-cell levels. This dearth can be recovered by the administration of polysaccharide A to germfree mice from the capsule of *Bacteroides fragilis*. This mechanism is conducted through pattern-recognition receptors (PRRs), such as toll- or NOD-like receptors, which recognize molecules that are induced by intestinal microbiota. These processes may attenuate certain inflammatory gut diseases by boosting the beneficial suppression of pathogenic bacteria, or regulating immune cells or PRRs [20,21]. In Crohn’s disease, individuals display mucosal dysbiosis, which may be pointed out by the reduced diversity of main microbes and *F. prausnitzii* [22,23,24,25,26,27,28,29].

Studies have suggested that *F. prausnitzii* contains an anti-inflammatory protein that inhibits the nuclear factor–κB (NF-κB) pathway in intestinal epithelial cells in animals. NF-κB represents a family of inducible transcription factors, that regulate a large number of genes involved in inflammatory processes. This family is composed of five structurally related members that mediate the transcription of target genes by binding to a specific DNA element, the κB enhancer, as various hetero- or homodimers [30]. Normally, NF-κB is locked in the cytoplasm by an inhibitory protein of the IκB family. Upon activation, IκB is phosphorylated by the IκB kinase complex (IKK), before undergoing degradation by the proteasome. Then, free NF-κB translocates to the nucleus to turn on a large number of genes involved in proinflammatory processes at the site of tissue damage [31]. Different studies have suggested that the gut microbiota plays a role in the inhibition of NF-κB [32,33]. Moreover, *Salmonella typhimurium* and *Clostridium difficile* utilize sialic acid, released by the gut microbiota, which favors their expansion in the gut [34,35,36,37,38,39]. The GI microbiota, through its metabolites, promotes the production of different antimicrobials, which include antimicrobial proteins (AMPs) such as cathelicidins and C-type lectins. Other mechanisms through which the gut microbiota can overcome pathogen growth are by promoting mucosal secretory IgA (SIgA). Moreover, pattern-recognition-receptor–microbe-associated-molecular-pattern (PRR–MAMP) interactions regulate several signaling tools that are crucial for promoting mucosal-barrier function and the production of AMPs, thus adding to host defense against pathogenic microbes [3,40,41,42,43].

### 1.2. Dysbiosis and Its Pathogenesis Factors

**Figure 1:**
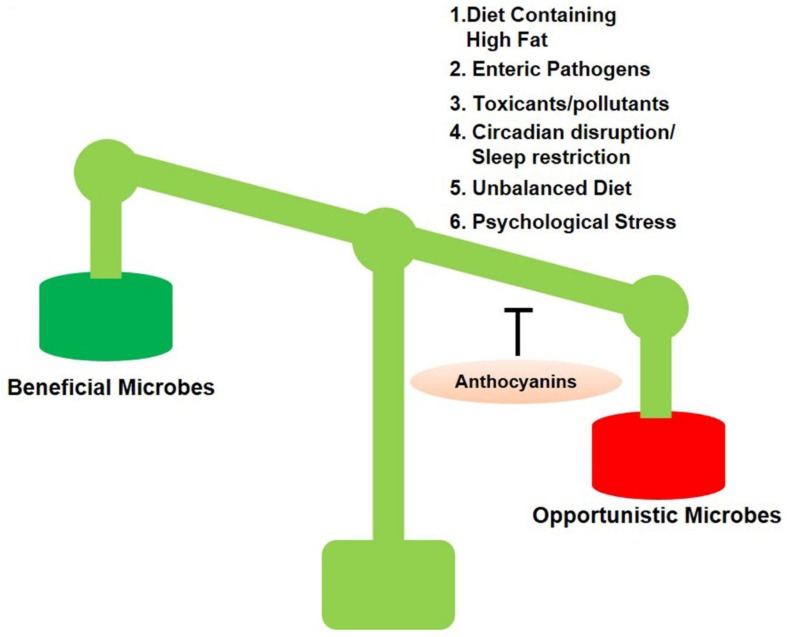
Anthocyanins switch population of opportunistic bacteria. Several factors provide opportunities to harmful microbes.

Dysbiosis is a term for a microbial imbalance inside the body, such as an impaired microbiota. There are many factors involved in dysbiosis that are given in Figure 1 [44].

#### 1.2.1. Congenital Factors

The microbial population can be affected by numerous factors, including mode of birth, nutrition, antibiotic exposure, stress, age, and degree of hygiene. For example, babies born through the vagina acquire the mother’s vaginal microbial flora, including Escherichia coli, Lactobacillus, Bifidobacterium, and Bacteroides. Those born via Cesarean section, on the other hand, have a high risk of skin-related bacterial invasion, including Staphylococcus species that continue throughout infancy [45,46,47,48,49,50,51,52].

#### 1.2.2. Dietary Factors

The effects of diet on the structure of gut microbes have been evaluated during the initial phase of colonization, as breastfed children have a greater number of *Bifidobacteria spp*, while formula-milk-fed children have higher levels of *Bacteroides spp*. Generally, a change in diet could induce 57% of total changes in gut microbiota, whereas congenital changes account for less than 12%, showing that diet has a prominent role in the structuring of the gut microbiota. The Western diet, which is rich in sugar and fat, causes dysbiosis, and affects the GI tract and the immune system [53,54,55]. The effects of a high-fat diet (HFD) on mice were also evaluated, where they were fed with a HFD for three months and showed a decline in the growth of *Bacteroidetes*, while the levels of *Firmicutes*, *Proteobacteria,* and *Actinobacteria* were markedly upregulated [56,57,58,59,60,61].

#### 1.2.3. Effects of Chemical Exposure on Gut Microbiota 

It was previously concluded that exposure to chemicals causes marked dysbiosis in the gut, which includes the augmented production of the Firmicutes phylum and reduced *Bacteroidetes* production. Similarly, the chronic administration of corticosterone causes gut dysbiosis. Furthermore, recent studies have suggested that the lack of intestinal alkaline phosphatase (IAP) induces dysbiosis, and elevates the inflammation and penetrability of the intestinal lining of newborns [62,63,64,65,66].

#### 1.2.4. Effects of General Stress on Gut Microbiota

Stress is a disturbance in body homeostasis, due to different kinds of factors, such as psychological and environmental stimuli, and physical stress, which provoke physiological and behavioral responses to reinstate homeostasis. Stress has a variety of biological effects, such as modulation of microbes in the GI tract. [67,68,69,70,71,72,73,74]. Here, we present recent studies associated with stress-induced changes in GI microbiota structure and composition. Stress types include sleep deprivation, psychological stress, circadian disturbance, environmental pathogens, environmental stress, pollutants, and diet, which were selected for their direct effect on environmental physiology and military personnel [75,76,77,78,79].

#### 1.2.5. Mental Stress and Gut Microbiota

Physiological stress may be induced by different means, including social deprivation and water restraint. These induce a disturbance in the body that causes inflammation, modulates immunity, alters GI function, and produces anxiety-like behaviors. From a military perspective, social defeat stress (SDS) is the most accepted model [80,81,82,83,84,85]. Using the SDS model in a study, 2 h SDS was enough to change mucosa-related microbes in mice, reducing *Lactobacillus reuteri*. When they were repeatedly exposed for 2 h over 6 days, this caused the higher attenuation of *Lactobacillus*. The relationship between gut, brain, and gut microbiota is bidirectional, where stress-triggered activation of the sympathetic nervous system (SNS) and the hypothalamic–pituitary–adrenal (HPA) axis affects gastrointestinal (GI)-tract microbes. Later, all these affect the central nervous system, either through the enteric nervous system, via spinal and vagal nerves, or via blood circulation when they gain entry to the blood stream and cross the blood–brain barrier [86,87,88,89].

#### 1.2.6. Altitude and Temperature Effects on Normal Gut Microbiota

It is commonly reported that the consequences of high altitude (2500 m) may affect the GI system (GIS). The symptoms that were observed because of hypobaric hypoxia are loss of appetite, indigestion, nausea, vomiting, gas, and abdominal pain. It has been reported that low oxygen delivery to the GI affects motility, because intestinal epithelia cells need oxygen-saturated blood for their normal physiological functions. An increase in the number of proinflammatory *Enterobacteriaceae* with increased inflammation and a decrease in the number of *Bifidobacteria* was found during a trip to Himalaya. Furthermore, it has been demonstrated that soldiers working or training at 3505 m altitude may decrease aerobic counts, while increasing beneficial and harmful micro-organisms [90,91,92,93,94,95].

A recent study showed that acute cold promotes alteration in murine gut microbiota [96,97,98,99,100]. Several studies indicated that the effect of cold on the human gut microbiota could be useful by promoting cold tolerance, which needs more exploration [101,102,103].

#### 1.2.7. Intestinal Infection (Enteric Pathogens) Accelerates Gut Dysbiosis and Alters Gastrointestinal-Wall Integrity 

A major type of diarrhea is called traveler’s diarrhea (TD), which commonly occurs in military personnel and is caused by enteroaggregative and enterotoxigenic E. *coli Salmonella spp, Shigella*, and *Campylobacter jejuni*. These agents cause diarrheal infections through the physical disruption of the gut barrier and immune-system disturbance, which leads to disturbing the gastric environment [104,105,106,107,108,109,110]. Some medically recommended antibiotics that are used for the treatment of infectious diarrhea and TD are azithromycin; levofloxacin enables the growth of opportunistic pathogens [111,112,113,114,115,116].

#### 1.2.8. Environmental Pollution and Toxins Affect Animal Health and Increase the Number of Opportunistic Gut Microbes 

Industrial chemicals and toxic industrial materials make the environment and air deleterious and unfit for survival. It is well known that burn pits are used to hide and destroy solid waste, particularly in military sites that carry toxic compounds, such as polycyclic aromatic hydrocarbons (PAHs), polychlorinated compounds, and particulates. A rodent model reported that exposure of adult mice to cadmium for 10 weeks changed gut-microbiota composition at the phylum and family levels. This same model also proposed that an increase in *Bacteroidaceae* boosts serum lipopolysaccharides (LPS). Moreover, it showed that the oral administration of B[a]P to experiment mice for 28 days resulted in moderate gastric inflammation and microbial-community shifts that included a decline in the level of anti-inflammatory taxa (e.g., *Lactobacillus* and *Akkermansia*), and an increase in the level of numerous inflammatory taxa [117,118,119,120]. 

#### 1.2.9. Effects of Noise on Gut Dysbiosis and Normal Microflora

When rodents were exposed to acoustic stress, intestinal tight-junction protein expression was decreased, intestinal permeability was increased, GI motility was changed, inflammation and intestine-tissue damage increased, and gastric-ulcer induction was observed. However, a study demonstrated that exposure of aged mice to severe noise for 4 h/day up to 30 days changed cecal microbiota, which was characterized by an increase in *Bacteroidetes/Firmicutes* ratio, associated with decreased expression of tight-junction proteins in the colon and hippocampus, inflammation, and Alzheimer’s-like cognitive impairments [121,122,123,124,125,126,127,128,129].

#### 1.2.10. Gut Dysbiosis Induces Neuroinflammation and Alzheimer’s Disease Pathology

**Figure 2:**
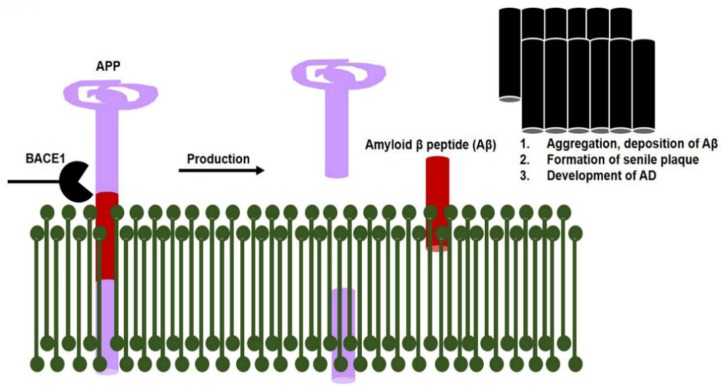
Increased beta-secretase 1 (BACE1) enzyme activity causes senile plaque formation. Increased BACE1 activity (when NF-κB transcription factor binds with its promoter region) causes abnormal APP cleavage that produces a burden of amyloid beta proteins. Consequences of amyloid beta plaques include Alzheimer’s disease development.

The lipopolysaccharides are secreted by gut bacteria during dysbiosis that exaggerate Alzheimer’s disease pathology, via the activation of amyloidogenic signaling pathways. Studies have suggested that bacterial-surface lipopolysaccharides bind with microglial receptors (TLR2, TLR4, and CD14) and activate the downstream NF-κB transcription factor. The activation of NF-κB producing proinflammatory cytokines that initiate neuroinflammatory responses. This neuroinflammatory response and reactive microglia activate various Alzheimer’s pathways, such as beta-secretase 1 (BACE1; Figure 2) [130,131,132,133,134,135,136]. 

However, a study revealed that lipopolysaccharides, endotoxins, and pathogens disrupt the gut and blood–brain barrier (BBB) tight junctions, and enter the brain, for example *Salmonella*, *E. coli,* and *Citrobacter* produce Aβ. In the brain, misfolding amyloid proteins may be triggered by an exposure to microbial communities. A prion-like mechanism is the same as the propagation and formation of Aβ seeds [134,137,138,139,140,141]. One possibility is that epithelial cells of mucosa-associated lymphoid tissue collect and pass it to the parasympathetic neurons of the vagus nerve and enteric nervous system, from where they may gain entry to the CNS through retrograde axonal transport. Dysregulation of these molecules can lead to neurotoxicity and chronic inflammation.

### 1.3. Daily Use of Natural Dietary Anthocyanins Increases Beneficial-Microbe Population, Prevents Leaky Gut, and Inhibits Circulatory Inflammagen (LPS) and Proinflammatory Cytokines

**Figure 3:**
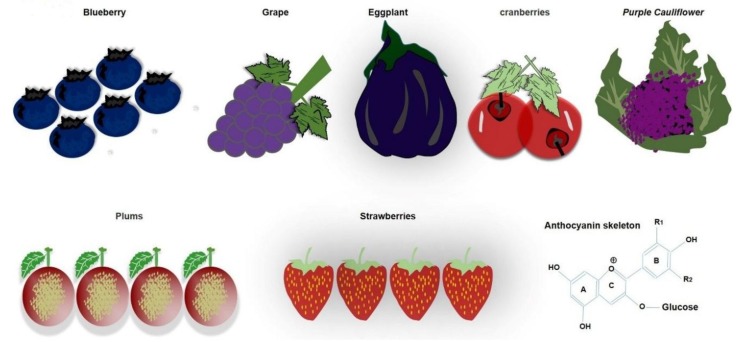
Natural dietary Anthocyanin sources. Various fruits and vegetables are rich sources of natural dietary anthocyanin.

Anthocyanins belong to flavonoids, are soluble in water, and are polyphenolic pigments that give color to food. There are some fruits and vegetables that are rich in anthocyanins, such as grapes, black plums, and blueberries, red and black rice, and black soybeans. The amount and composition of anthocyanins in different fruits and vegetables range from 0.1% to 1% (Figure 3). 

Berries are a rich source of anthocyanins, so they may help in different functions of the body, as highlighted previously [142,143].

**Figure 4:**
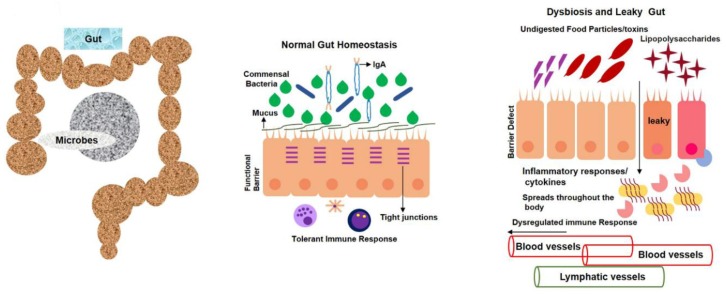
Leaky gut contributes to circulatory inflammagens and proinflammatory cytokines. Opportunistic bacteria produce various toxins and polysaccharides that, in turn, cause intestinal-wall inflammation that results in a leaky gut. Epithelial-cell inflammation also leads to loss of tight junctions that provide opportunities for undigested food particles, toxins, and inflammatory cytokines to enter bloodstream. Toxins and lipopolysaccharide (LPS)-containing blood later enter brain via blood–brain-barrier disruption.

Furthermore, enzymolysis, oxidation, and climatic factors like light, temperature, and pH can change the level of anthocyanins. Acidic conditions are better for the stability of anthocyanins. In most cases though, it is degraded at high pH. Anthocyanins are naturally present in plants as glycosides carrying glucose, galactose, arabinose, rhamnose, and xylose [144,145,146,147,148,149,150,151,152,153]. The unstable form of deglycosylated anthocyanins is called anthocyanidins, which are very rare in nature. The peculiar electron distribution and presence of flavylium ion are the reasons for anthocyanidin instability. On the basis of chemical structure, 700 anthocyanins and 27 aglycones have so far been recognized. The basic structure of anthocyanin comprises a C-6 (A ring)-C-3 (C ring)-C-6 (B ring) carbon skeleton with different numbers of sugars and hydroxyl groups, and varying degrees of methylation [144,146,153,154,155,156,157]. It was established that tight junctions provide a paracellular barrier that only allows select molecules into the intercellular space between epithelial cells (Figure 4). 

Cellular adhesion occurs because of occluding-regulated paracellular permeability. A classic TJ marker, ZO-1, works as an anchor and contributes to connecting occludin, claudin, and actin cytoskeletons to increase the epithelial barrier [158,159,160,161,162,163,164,165,166,167,168,169]. An in vivo study was carried out on rodents, where adult mice received 100 mg/kg black-rice extract per os (P.O.) for five days, in order to induce colitis, which indicated that mice that received black-rice extract had fewer histological lesions in their mucosa, as compared to dextran sodium sulfate-treated mice. It was suggested that anthocyanins prevent starch digestion by inhibiting the ά-amylase enzyme. When this undigested starch reaches the large intestine, it provides energy to probiotic bacteria, including *Lactobacilli, Bifidobacteria, and Streptococci*, which later on improves health conditions [167,170,171,172,173,174,175,176,177].

### 1.4. Anthocyanins Mitigate Gut Dysbiosis that Induces Neuroinflammation and Alzheimer’s Pathology

**Figure 5:**
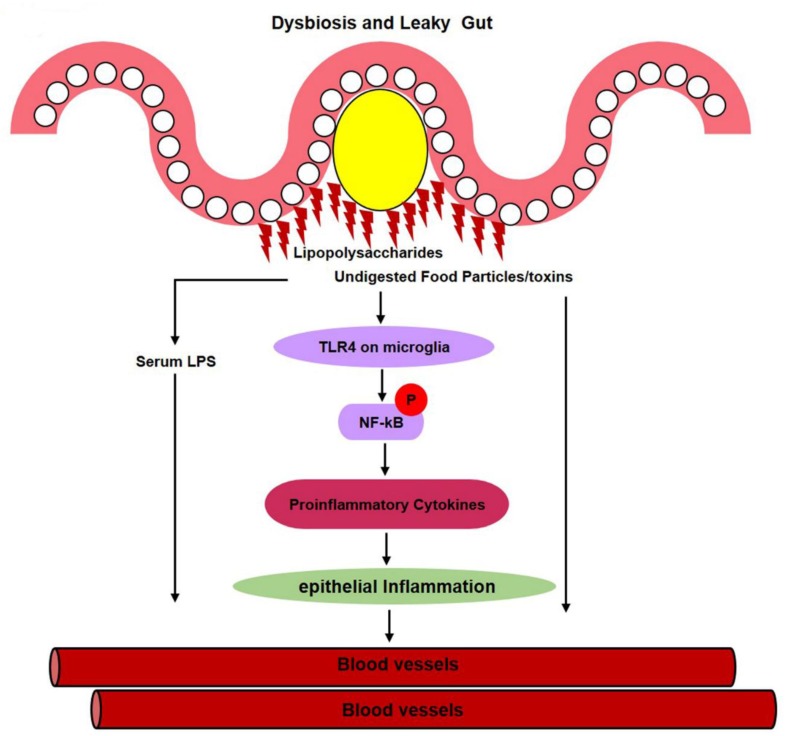
Gut dysbiosis causes epithelial inflammation. When harmful, particularly *E. coli*, Gram-negative bacteria secrete lipopolysaccharides that activate the TLR4 pathway and later on cause intestinal-wall and epithelial inflammation. This lipopolysaccharide not only activates the TLR4 signaling pathway, but also microglial and astrocyte cells in the gut, that then secrete proinflammatory cytokines. These proinflammatory cytokines later gain entry to the bloodstream via a leaky gut. This serum LPS then disrupts the blood–brain-barrier (BBB) and enters the brain, where it reactivates microglia, and various inflammatory and amyloid genic pathways.

It was reported that LPS can enter the bloodstream via a damaged intestinal epithelium during an enteric-dysbiosis failure. The increase in the abundance of opportunistic pathogens could cause an increase in serum LPS levels. LPS further activates inflammatory pathways, including TLR4/NF-κB, which, in turn, leads to the release of proinflammatory cytokines, such as TNF-ά, IL-1β, and CO_2_, that then enter the bloodstream via a leaky gut (Figure 5) [178,179,180]. 

Previous studies reported that the abnormal cleavage of the amyloid beta protein by β-secretase results in amyloid beta plaque formation. Increased amyloid beta plaques accelerate neuronal cell death and Alzheimer’s pathology [181,182,183]. Moreover, gut bacteria populating the microbiome were shown to produce amyloids and other immunogenic mediators that contribute to the modulation of signaling mechanisms implicated in neuroinflammation, brain Aβ deposition, and AD pathogenesis [184]. To explain how gut microbiota might contribute to AD pathogenesis, it was hypothesized that bacteria-derived amyloids leak from the gastrointestinal tract and accumulate at system and brain levels [185]. 

**Figure 6:**
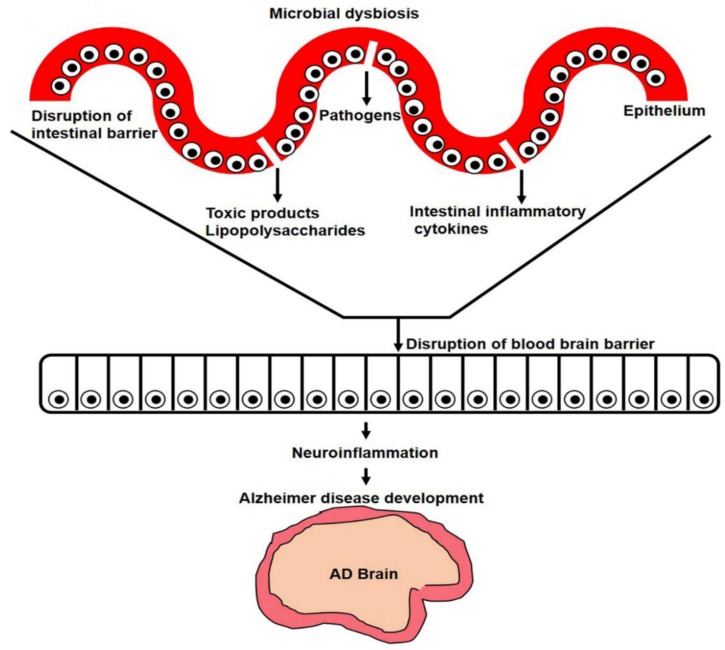
Gut-microbiota-secreted polysaccharides and other toxins cause neuroinflammation and Alzheimer’s disease development. When circulatory lipopolysaccharides enter the brain, it activates inflammatory pathways and increases the number of reactive microglia. Increased inflammatory cytokines and NF-κB increase APP and amyloid beta protein cleavage and accumulation, and cause Alzheimer’s disease development.

Here, we summarized the neuroinflammation-mediated amyloid beta burden and Alzheimer’s pathology because, in dysbiosis, the opportunistic bacterial component (LPS) is prominently generated inside the gut, which later gains entry to the brain via the blood and causes a neuroinflammation-mediated amyloid beta burden (Figure 6) [186,187].

**Figure 7:**
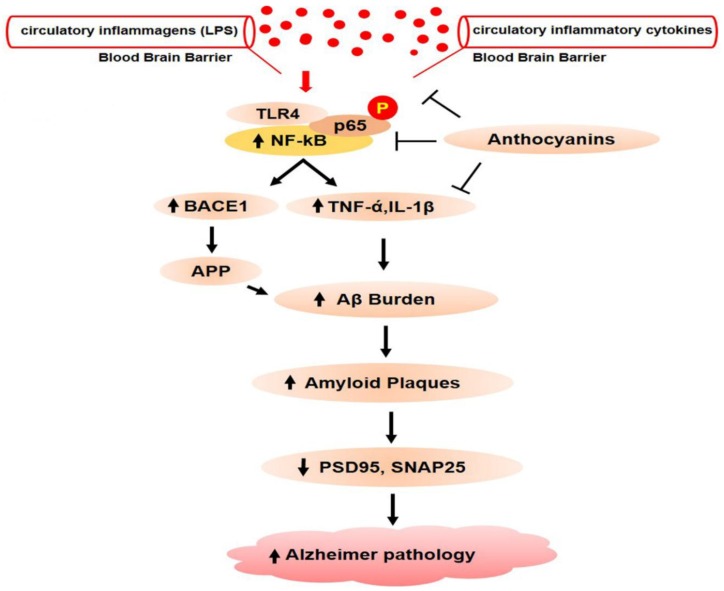
Anthocyanins prevent inflammatory and amyloid genic pathways in the central nervous system. When circulatory lipopolysaccharides and inflammatory cytokines gain entry to the brain, toll-like receptor 4 on microglia is activated, which subsequently leads to the activation of nuclear factor kappa B (NF-κB). After NF-κB phosphorylation and translocation to the nucleus, it goes on to bind with proinflammatory cytokine genes, that results in the production and release of proinflammatory cytokines. NF-κB also binds with the promoter region of BACE1 that produces β-secretase enzymes. Increased BACE1 activities later result in abnormal APP cleavage. Chronic deposition of amyloid beta proteins in the brain causes amyloid beta plaque formation and neuronal cell death. Amyloid beta plaques also deregulate pre- and postsynaptic proteins, which results in dementia and memory impairment.

It has been reported that LPS is the ligand of the TLR4 receptor that exists on microglia cells. When LPS bind with TLR4, it activates the inflammatory cascade by translocating the downstream NF-κB to the nucleus. Once NF-κB phosphorylates, it binds with proinflammatory cytokine (TNF-ά, IL-1β, and COX2) as a result, increases the neuroinflammation (Figure 7). 

Ali T and Khan, M.S. et al. recently reported that anthocyanins could significantly ameliorate the expression of proinflammatory cytokines and ROS/JNK, thus preventing neuroinflammation and Alzheimer’s pathology [188,189,190,191,192,193,194,195,196,197,198]. Moreover, a study has shown the rescuing effects of anthocyanin against Alzheimer’s disease pathology (APP, BACE-1, Aβ, and P-tau) and synapsis-related functions in Aβ1-42-injected mice [199]. The effects of anthocyanin against gut-dysbiosis-induced neuroinflammation are in accordance with previously conducted studies, showing that nutrients have a significant effect against microbiota-induced neurocognitive disorders [200].

## 2. Conclusions and Future Perspectives

**Figure 8:**
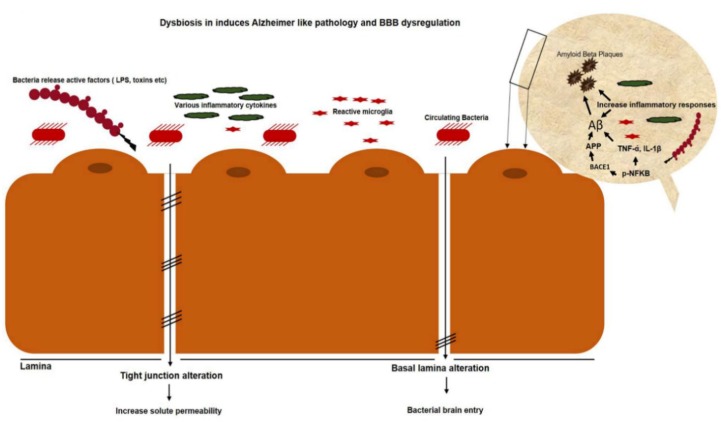
Circulatory toxins and lipopolysaccharides cause blood–brain barrier (BBB) disruption. Circulatory-microbe-secreted toxins, lipopolysaccharides, pathogens, and proinflammatory cytokines disrupt the blood–brain barrier and enter the brain, which activates various apoptotic, inflammatory, and amyloid genic pathways.

In our study, we focused on gut dysbiosis that induces systemic toxins, inflammagen-mediated neuroinflammation, and Alzheimer’s pathology. We summarized that gut dysbiosis not only induces gastrointestinal disorder, particularly epithelial inflammation, tight-junction disruption, and leaky gut, but also contaminates the circulatory blood that results in BBB disruption, neuroinflammation-mediated Alzheimer’s pathology, and memory dysfunction (Figure 8). 

There are a number of signaling pathways that are involved in systemic inflammagen-mediated neuroinflammation and Alzheimer’s pathology. However, we focused on the TLR4/NF-κB, ROS/JNK, and NF-κB/BACE1 pathways, and proposed that targeting these pathways by natural dietary polyflavonoid anthocyanins not only abrogates gut-dysbiosis-induced inflammagen (LPS, proinflammatory cytokines, toxic amines)-mediated neuroinflammation and Alzheimer’s pathology, but also provides opportunities for clinical and preclinical studies in the future.
